# An Aggressive Approach to Locally Confined Pancreatic Cancer: Defining Surgical and Oncologic Outcomes Unique to Pancreatectomy with Celiac Axis Resection (DP-CAR)

**DOI:** 10.1245/s10434-020-09201-2

**Published:** 2020-10-13

**Authors:** Ryan K. Schmocker, Michael J. Wright, Ding Ding, Michael J. Beckman, Ammar A. Javed, John L. Cameron, Kelly J Lafaro, William R. Burns, Matthew J. Weiss, Jin He, Christopher L. Wolfgang, Richard A. Burkhart

**Affiliations:** 1. The Division of Hepatobiliary and Pancreatic Surgery, Johns Hopkins Hospital, Baltimore, MD; 2. The Division of Surgical Oncology, Donald and Barbara Zucker School of Medicine at Hofstra/Northwell, New Hyde Park, NY

## Abstract

**Background:**

Modern chemotherapeutics have led to improved systemic disease control for patients with locally advanced pancreatic cancer (LAPC). Surgical strategies such as distal pancreatectomy with celiac axis resection (DP-CAR) are increasingly entertained. Herein we review procedure specific outcomes and assess biologic rationale for DP-CAR.

**Methods:**

A prospectively maintained single-institution database of all pancreatectomies was queried for patients undergoing DP-CAR. We excluded all patients for whom complete data were not available and those who were not treated with contemporary multi-agent therapy. Data was supplemented with dedicated chart review and outreach for long-term oncologic outcomes.

**Results:**

Fifty-four patients underwent DP-CAR between 2008–2018. The median age was 62.7 years. 98% received induction chemotherapy. Arterial reconstruction was performed in 17% and concomitant visceral resection in 30%. R0 resection rate was 87%. Postoperative complications were common (43%) with chyle leak being the most frequent (17%). Length of stay was 8 days, readmission occurred in one-third, and ninety-day mortality was 2%. Disease recurrence occurred in 74% during a median follow up of 17.4 months. Median recurrence-free (RFS) and overall survival (OS) were 9 and 25 months, respectively.

**Conclusions:**

Following modern induction paradigms, DP-CAR can be performed with low mortality, manageable morbidity, and excellent rates of margin-negative resection in high volume settings. The profile of complications of DP-CAR is distinct from pancreaticoduodenectomy and simple distal pancreatectomy. OS and RFS are similar to those undergoing resection of borderline resectable and resectable disease. Improved systemic disease control will likely lead to increasing utilization of aggressive surgical approaches to LAPC.

## Introduction:

Pancreatic cancer remains a devastating disease and currently represents the fourth leading cause of cancer-related deaths in the United States.^1^ In 2020, an estimated 57,600 new pancreatic cancer cases and 47,050 pancreas cancer related deaths are expected.^1^ Unfortunately, the majority of patients present with metastatic (50%) or locally advanced disease (30%), leaving only 20% of patients that are deemed resectable at the time of presentation.^2^ Surgical extirpation remains the only chance for cure in this disease. A subset of locally advanced disease (stage III) tumors are considered unresectable due to the involvement of critical locoregional vascular structures, including more than 180 degree involvement of the celiac axis (CA) or superior mesenteric artery (SMA).^3^ As systemic therapies improve, surgical oncologists now recognize that there is a subset of patients that are characterized by locally extensive primary tumors in the absence of clinically apparent metastatic disease (historically up to 30% of patients with stage III disease).^4^ As systemic disease control improves, more aggressive surgical approaches are being entertained and the nomenclature is changing around this subset of patients with locally confined disease. Recent reports have demonstrated margin negative resection is possible in this locally confined cohort, with rates up to 69% following induction chemotherapy.^5^

The Appleby procedure, originally described in 1953, was utilized for locally advanced gastric cancer with involvement of the CA.^6^ Complete tumor extirpation is achieved with *en bloc* resection of the celiac axis and its proximal branches. Preservation of hepatic arterial perfusion remains via retrograde flow from the SMA though the gastroduodenal artery (GDA) and to the proper hepatic artery.^6^ The original technique was subsequently applied to cancer of the pancreatic body and tail,^7^ and ultimately modified for gastric preservation with retention of the native right gastric and, at times, right gastroepiploic arterial cascades.^8^ This operation is gaining favor in high-volume centers for appropriately selected patients with biologically favorable locally confined pancreatic cancer and carries two names, the modified Appleby procedure or distal pancreatectomy with *en bloc* celiac axis resection (DP-CAR) for pancreatic cancer. Over the last several years there have been increasing reports of application of DP-CAR for pancreatic adenocarcinoma which have demonstrated acceptable morbidity and mortality, with improved survival when compared with historic controls of patients treated with chemotherapy alone.^9,10^ However, details regarding procedure specific complications and oncologic rationale remain sparse in the literature. The objective of this study was to examine the complications for patients with pancreatic adenocarcinoma undergoing DP-CAR and to evaluate the oncologic rationale for resection through the study of patient outcomes and patterns of recurrence.

## Methods:

### Study Population:

A prospectively maintained single institution database of all pancreatic resections was queried for patients older than 18 years undergoing distal pancreatectomy with arterial resection/reconstruction for pathologically confirmed pancreatic adenocarcinoma. Extensive chart review was performed to enhance the capture of relevant detail and to abstract clinical variables. Demographic, procedure specific, and pathologic variables were collected. In addition, complications, patterns of recurrence, and long-term outcome data were gathered. Mortality data were supplemented by state death records and a search of the social security death index (SSDI). Patients undergoing surgery from 2008–2018 were included. Data were collected according to guidelines outlined and approved by our institution’s ethical review board.

### Outcomes:

Postoperative liver function tests were reported as the values obtained on the postoperative day 1. Chyle leak was defined as output of milky-colored fluid from a drain, drain site, or wound on or after postoperative day 3, with a triglyceride content more than 110 mg/dL.^11^ Clinically relevant postoperative pancreatic complications were defined according to the definitions outlined by the International Study Group (ISGPS) for pancreatic fistula and delayed gastric emptying.^12,13^ Overall complication rates were collected and graded according to the Clavien-Dindo Classification.^14^ Pathological response to induction/neoadjuvant chemotherapy was reported per the College of American Pathologists (CAP) guidelines.^15^ Overall survival was calculated from the date of surgery to the date of death, and censored at the date of last follow up for patients found to be alive at most recent follow up. Unique gastric complications, such as ischemia or perforation, were identified on cross sectional imaging, re-intervention or re-operation. Positive lymph node ratio is defined as ratio of positive lymph nodes to all lymph nodes undergoing pathologic examination. An R1 resection was defined as malignant cells within 1mm from the final margin. Recurrence-free survival was calculated from the date of surgery to the date of first radiographic evidence of recurrence, metastatic disease or censored at the date of last follow up for patients found to have no evidence of disease on most recent follow up.

### Patient Selection and Technical Details:

The clinical patient assessment included a history, examination and high-quality 3D computed tomography assessment. All patients were assessed in a multidisciplinary setting. In keeping with national and institutional standards for care, those with borderline resectable pancreatic cancer (BRPC) or locally advanced pancreatic cancer (LAPC) on initial assessment were referred for systemic therapies with an eye towards a neoadjuvant approach (for BRPC) or induction chemotherapy (for LAPC) often with preoperative utilization of radiotherapy.

DP-CAR was approached in a manner similar to that previously described.^16^ Briefly, after ruling out disease dissemination the anterior aspect of the pancreas is exposed by raising the omentum off of the transverse mesocolon in the avascular plane thereby preserving the gastroepiploic arterial arcade. The common hepatic artery (CHA) is encircled proximal to the GDA takeoff with caution to preserve both the GDA and the right gastric artery. The capacity for GDA backflow into the proper hepatic artery is verified by palpation and doppler ultrasonography after test clamping of the CHA for approximately 5 minutes. If the pulse is lost, an intraoperative assessment of the patient’s appropriateness for arterial revascularization is performed. Otherwise, the case proceeds with dissection of the supra-celiac aorta and identification of the celiac takeoff. With a suitable cuff of the CA identified the case proceeds with transection of the pancreatic neck, division of the major vascular structures and standard en bloc resection with distal pancreatectomy and splenectomy. Early in our experience only patients without extensive mesenteric vein involvement were explored. For example, those with limited and/or short segment venous involvement were candidates for resection, however for those who exceeded the capacity to be reconstructed in ‘traditional’ fashion (longitudinal venous repair, patch repair, or short segment end-to-end) the procedure was terminated. More recently we have pushed further into utilization of extra-anatomic venous bypass techniques, mesocaval/mesoportal shunting to facilitate complete tumor extirpation.

### Statistics:

Non-normally distributed continuous variables were examined using non-parametric methods. Progression-free survival and overall survival were estimated using Kaplan-Meier methods. Statistical analyses were performed using SAS 9.4 (SAS Institute Inc., Cary, NC, USA).

## Results:

### Preoperative Clinical Findings and Treatment Course

**Figure 1 –:**
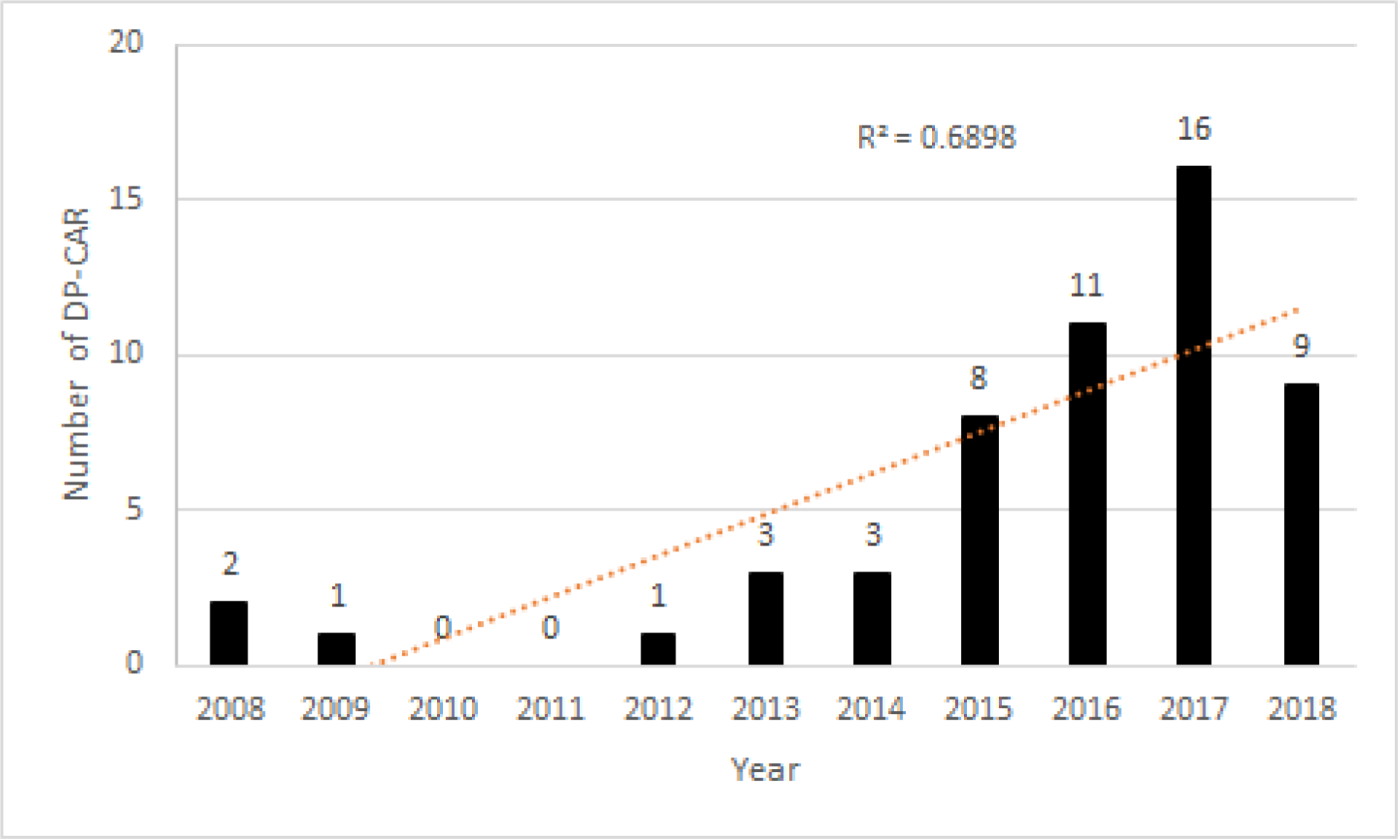
Trends in the number of DP-CARs performed per year: Examination of our experience demonstrates significantly increased utilization of DP-CAR over the duration of the study period, with the vast majority performed in the last 4 years of the study.

Fifty-four patients underwent DP-CAR for PDAC during the ten-year defined period. In addition, the number of DP-CAR cases performed at our institution has increased over time (R^2^ = 0.690, p < 0.001, Figure 1). The median follow-up for clinical and oncologic data was 17.4 months (IQR: 9.8–27.6). The median age was 62.7 (IQR: 57.0–68.0), with 46.3% male (Table 1). The majority of patients were Caucasian (85.2%) and most carried additional comorbid conditions attributing to mild (ASA II – 20.4%) or severe systemic disease (ASA III – 72.2%). The most frequent comorbidity was hypertension (48.2%), and 11.1% and 18.5% of patients were currently smoking and using alcohol, respectively. The majority of patients were symptomatic on presentation, with abdominal pain being reported most commonly (77.8%). CA19–9 was elevated in 63.0% (n=34) preoperatively, with a median of 69.0 U/mL (IQR: 33.5–370.1). Four patients were non-expressors of the Lewis Antigen. CA19–9, while often assessed as a trend and used as a prognostic biomarker of chemotherapeutic response in the induction setting, was not used independently as a rationale to either offer or restrict a surgical approach. Practically, those with rising CA19–9 values during the induction period frequently developed additional sites of disease prior to surgical intervention and were not captured in our surgical database. Preoperative assessment of both liver function (bilirubin) and kidney function (creatinine) was within normal limits for almost all patients (100% and 96.3%, respectively).

All but one patient (unanticipated arterial involvement based on preoperative cross-sectional imaging) were staged as locally advanced on presentation and received preoperative chemotherapy, with most receiving FOLFIRINOX (70.4%) or Gemcitabine/nab-paclitaxel (20.4%, Table 1). The median duration of induction chemotherapy was 4.0 months (IQR: 4.0–6.0). Radiation therapy was included in the preoperative treatment paradigm for 51 of 54 patients, with stereotactic body radiation therapy (SBRT) being the most commonly utilized modality (74.1%). At the completion of induction treatment gross radiographic tumor stability was demonstrated in 62.9% (n=34), reduced tumor size in 24.1% (n=13), and locally progressive disease in 5.6% (n=3).

### Operative Characteristics

The median operative time in this cohort was 371 min (IQR: 281 – 432), with a median blood loss of 750 mL (IQR: 400 – 1000 mL). Arterial reconstruction was performed in 16.7% with the majority using prosthetic material as conduit (7/9). Autologous saphenous vein and cryopreserved artery were each used in one patient. The majority of reconstructions were jump grafts from the celiac artery stump or aorta to the CHA (6/9). The remaining jump grafts were from the iliac to CHA, a bifurcated graft from the iliac to right and left hepatic arteries, and right renal artery to CHA (Table 2). Three of the patients who required arterial reconstructions had preoperative findings that indicated the potential need for reconstruction. The remaining 6 patients had diminutive retrograde flow through the GDA found intraoperatively after test-clamping of the CHA. Intraoperative assessment of the GDA flow to the Proper Hepatic Artery was assessed most commonly by (1) direct palpation, (2) doppler ultrasonography, or (3) backbleeding after sharp transection of the CHA.

Multivisceral resection was performed in 29.6% of patients with partial or total gastrectomy for tumor involvement being the most common (n=10), followed by partial or total left adrenalectomy (n=7), left nephrectomy (n=1) and liver wedge resection (n=1, Table 2). 29.6% of patients received a blood transfusion in the perioperative period. Most commonly, partial gastrectomy was performed after tumor extirpation and at the discretion of the operating surgeon upon noting compromised arterial inflow or venous drainage. While objective intraoperative data regarding the adequacy of arterial inflow were not obtained, most were felt to be required due to venous congestion and compromised outflow. In one patient, elective gastrectomy was preplanned as the patient had prior antrectomy with vagotomy for ulcer disease compromising the right gastric and right gastroepiploic cascades.

### Postoperative and Pathologic Outcomes

Median length of stay was 8.0 days (IQR: 6.0–9.0). The perioperative hepatic insult, as measured by postoperative transaminases, was mild in this cohort (median ALT 96U/L (IQR: 42 −239), AST 104U/L (IQR: 55–175)). No patients experienced post-operative liver failure. Complications occurred in 42.6% of patients with chyle leak being the most commonly encountered (16.7%, Table 3). All chyle leaks were low volume (less than 300mL daily output), which were self-limited and treated with low fat diets and prolonged abdominal drainage. All but one patient with chyle leaks retained their surgically placed drain at the time of discharge. No patient required additional percutaneous drain placement, octreotide, or parental nutrition secondary to the chyle leak. Pancreatic fistula occurred in 9.3%. Despite attention paid to the preservation of right-sided gastric vascularization, one patient developed a gastrocutaneous fistula and one in our early experience developed gastric ischemia and perforation requiring re-exploration and partial gastrectomy. There were no thirty-day mortalities, however ninety-day mortality occurred in one patient secondary to an ischemic stroke on postoperative day 65.

R0 resection was achieved in 87.0%, with 35.2% patients having at least 1 positive node (Table 2). Lymphovascular invasion was uncommon (24.1%), whereas perineural invasion was seen in the majority of patients (57.4%). For the patients receiving induction therapy, 48.1% (26/54) had invasion of the CA or CHA (ypT4) noted on final pathology. Most patients had moderately differentiated tumors (56.6%). Preoperative treatment response was reported in 83.3% (45/54), with 31.1% having a near complete response (CAP Grade 1), 51.1% partial response (CAP Grade 2), and 17.8% poor or no response (CAP Grade 3). There were no complete responses in this subset (Table 2).

### Long Term Outcomes

**Figure 2 –:**
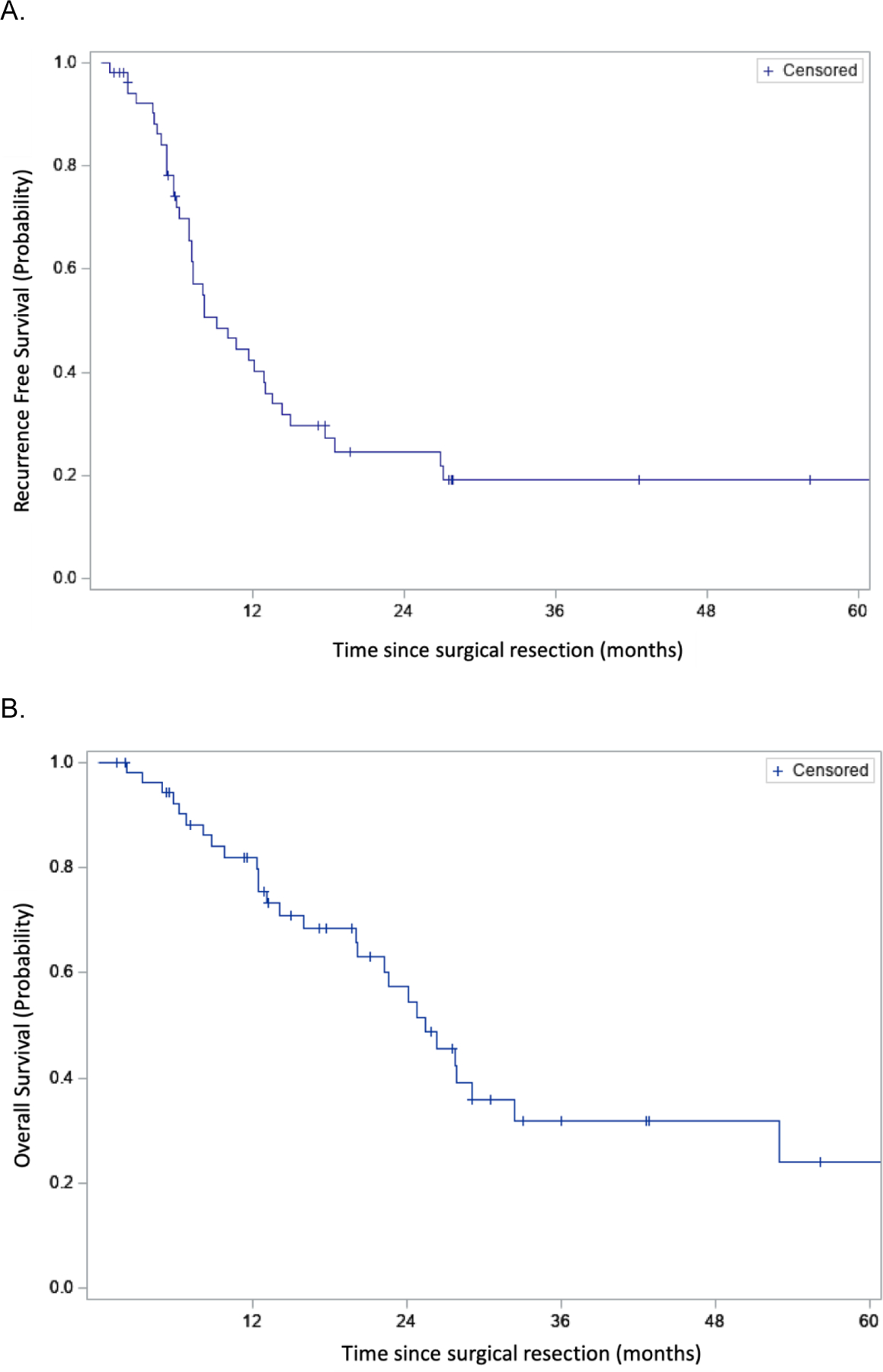
Survival Outcomes: Kaplan-Meier curves of (A) Recurrence Free Survival (median: 9.1 months; IQR: 7.1–13.0) and (B) Overall Survival of the entire cohort (median: 25.4 months; IQR: 20.2–32.4).

Adjuvant therapy data were not available for 10 patients. Of those that had available data, 61.4% (27/44) received further adjuvant therapy, for a median duration of 3.0 months (IQR: 2.0–5.0). The most common reasons for not initiating therapy were postoperative complications (n=7) and additional therapy was not recommended by primary oncologist and surgical oncologist (n=5). Standard follow-up was performed for patients undergoing DP-CAR and included assessment of tumor markers and surveillance cross-sectional imaging every 3 months for the first two years, every six months in years 3–5, and then annually thereafter. During routine follow-up, 74.0% of patients recurred with a median follow up of 17.4 months (IQR: 9.8–27.6). Local recurrence alone was the most common site of recurrence (35.1%), followed by lung only (13.0%) and peritoneal disease (11.1%, Table 4). Interestingly, liver only recurrence was uncommon (7.4%). Median recurrence-free survival was 9.1 months (IQR: 7.1–13.0) and overall survival was 25.4 months (IQR: 20.2–32.4, Figure 2).

## Discussion:

Improvements in systemic chemotherapy, and therefore systemic control, for patients with pancreatic adenocarcinoma have opened the door for aggressive surgical therapies for patients with locally aggressive primary tumors in the absence of systemic disease. This is a subset of patients that, in the past, were not surgical candidates and whose outcomes were characterized by poor overall survival with chemotherapy alone (median 8–12 months).^17–28^ Examination of our experience – the largest single-center Western experience to date – has demonstrated that patients undergoing aggressive surgical therapy with celiac resection have a reasonable postoperative course with acceptable morbidity and mortality. DP-CAR is associated with a unique profile of postoperative complications (chyle leak and gastric ischemia/perforation) compared with our experience of patients undergoing distal pancreatectomy and splenectomy alone. In addition, patterns of disease recurrence after DP-CAR tends more towards local or peritoneal failure as compared to the frequent hepatic failure seen following a pancreaticoduodenectomy or distal pancreatectomy. We conclude that there is a justification of this aggressive approach based on the survival outcomes as compared with historic controls of patients with locally advanced pancreatic adenocarcinoma treated with chemotherapy alone. This work specifically demonstrates relatively low mortality and manageable morbidity when DP-CAR is completed in a very high-volume setting.

In our study group we found an overall complication rate of 42.6% and serious complication rate (Clavien-Dindo ≥3) of 18.5%. Ninety-day mortality was 1.9%. These data are comparable with other recent reports on DP-CAR. For example, a recent systematic review of the literature identified 240 patients in 19 studies and reported a major morbidity of 27% and 90-day mortality of 3.5%.^30^ In a multicenter European study, 191 DP-CAR patients were identified and the overall 90-day mortality rate was 9.5%, with a mortality rate of 5.5% in high-volume (1 ≥ DP-CAR/year) centers and 18% at low-volume centers.^9^ Other single-center studies reported 90-day mortality rates as high as 17 and 18%.^31,32^ Even large database studies, such as a NSQIP analysis showed a 10% mortality rate and 10% incidence of acute kidney injury.^33^ Morbidity rates have also varied widely in published literature, with one reporting a rate of 92%.^20^ Taken together, these data support the assertion that outcomes are dependent largely on patient selection and suggest that a volume-outcome curve may exist for this complex procedure.

Given the granularity of our data we were able to examine the specific complications that contributed to morbidity in our cohort. An interesting observation was the high proportion of patients that had a postoperative chyle leak (16.7%), which is much higher than a previous report at our institution. In our overall experience, 1.3% of all pancreatectomies demonstrated a chyle leak or chylous ascites, and only one patient had a chyle leak in 711 distal pancreatectomies.^34^ A more recent single institutional study demonstrated a chyle leak rate of 10.4% in all patients, 15.1% in distal pancreatectomies, with distal pancreatectomy being a significant predictor (p=0.001) of chyle leak development on multivariate analysis.^35^ The explanation for increased chyle leak in patient undergoing DP-CAR is likely related to the extensive dissection along the aorta and SMA to expose the tumor and celiac axis. There is a rich lymphatic network in this area converging at the cisterna chyli, which is in close proximity to the planned dissection plane. Further, complications such as hepatic and gastric ischemia are also unique to celiac axis resection when compared with a standard distal pancreatectomy. As discussed previously, ensuring adequate liver perfusion after resection of the celiac axis is of utmost importance as hepatic artery ischemia is a devastating complication. Fortunately, in our study, we had no patients that had complications related to hepatic artery ischemia and LFTs were modestly elevated at medians of 96.0U/L and 104.0U/L for ALT and AST, respectively on postoperative day one. We are liberal in performing arterial reconstruction in patients with concern for hepatic malperfusion, likely explaining our favorable outcome compared with other reports. For example, others have reported liver ischemia rates ranging from 18–21%, without improvement in ischemic complication with preoperative hepatic artery embolization,^9,36^ perhaps again highlighting the importance of thoughtful patient selection. In addition, gastric ischemia is a well described consequence of celiac axis resection, and ensuring adequate gastric perfusion prevents the downstream morbidity and mortality of gastric perforation, ischemic gastropathy, and refractory ulcer disease. In our study we had 2 patients (3.7%) with ischemic gastric complications, one requiring reoperation and managed with total gastrectomy and the other undergoing percutaneous drain insertion and unplanned IV antibiotic therapy. This is in line with previous reports. For example, Klopmaker *et al.* reported several patients (18 patients) with ischemic gastric complications, with 4 serious complications (necrosis and ulcer disease).^30^ Another group had a 29% rate of ischemic gastropathy.^37^ Gastric ischemic ulcer disease leading to death has also been reported.^32^

We also examined the oncologic benefit for those undergoing DP-CAR through the lens of the patterns of recurrence for this cohort. We found that 74.0% of patients recurred during the follow up period. This rate of recurrence is similar to that for patients undergoing other types of partial pancreatectomy for PDAC.^38,39^ The ESPAC-4 follow-up data included 60 patients undergoing distal pancreatectomy. One-third developed local only recurrence, 28% having distant only recurrence, and the liver being the most common site of distant disease recurrence.^39^ Local recurrence was most common in our study (35.1%), and our patients had infrequent liver only disease (7.4%). Of note, only 20.4% had distant only disease at the time of recurrence. Peritoneal disease alone is not clearly reported in other studies, but a large proportion of our patients had peritoneal only disease recurrence (11.1%). Patients with pancreatic head cancer tend to have an increased incidence liver only disease (25%), with a similar proportion of local only recurrences (24%).^38^ We hypothesize that the patients undergoing DP-CAR may represent a unique subset of locally aggressive tumors that, after extensive courses of induction chemotherapy and radiotherapy, may have a reduced propensity for liver metastases. This is likely not unique to the tumor location or nature of the operation, rather the highly selective process to which this cohort was subjected.

As the vast majority of our patients received preoperative chemotherapy and radiotherapy, we examined the margin negative resection rates and survival outcomes of this aggressive approach. We observed an R0 resection rate of 87.0%, which compares favorably with other large reports of patients undergoing DP-CAR (31–93%),^9,20,30,36,40–42^ and is likely a result of our approach to patient selection. Our cohort also demonstrated favorable median recurrence-free (9.1 months) and overall survival (25.4 months) compared with other cohort studies. This association with relatively favorable oncologic outcomes is echoed in several other studies where neoadjuvant and induction chemotherapy was associated with significant improvement in overall survival.^30,31,43,44^ In the Japanese experience, unprecedented levels of survival have been seen (31–40 month, overall survival time), however the high usage rates and response to S-1 suggests this represents a may be a unique patient cohort.^43,44^ Other studies with less heavily pre-treated patients demonstrated survival rates from 12–30.9 months.^9,30,32,37^ Finally, one small study which examined patients undergoing DP-CAR as compared with chemotherapy alone suggested that surgical extirpation of the primary tumor is associated with an improvement in overall survival (Median OS DP-CAR vs. Chemotherapy; 20.8 vs 9.8 months; P = .01),^20^ adding further evidence to an aggressive surgical approach for patients who may be otherwise unresectable due to locally aggressive disease.

This work has several limitations which should be kept in mind when interpreting our experience. First, we present highly annotated data from a relatively limited number of patients treated at a single high volume academic medical center. This clearly limits the generalizability of both the surgical technique and observed outcomes. In addition, the retrospective nature of this study is prone to bias. We have additionally utilized stereotactic body radiation therapy liberally in this cohort, often calling on our high-volume pancreas specific radiation oncologists to map dose volumes to high-quality 3D scans. This utilization may additionally limit generalizability to other centers. Also, it is important to put this experience in context with the common contraindications we utilized when selecting patients for surgery. The principle factors preventing patients from undergoing surgical exploration were: (1) the emergence of overt metastatic disease during the induction phase of therapeutic administration, (2) comorbid medical risks which were assessed to be prohibitive when taken in the context of a surgical strategy targeting locoregional disease, (3) early in the experience we declined to explore patients with both celiac involvement and portomesenteric axis involvement in a manner not amenable to standard reconstruction techniques, or (4) a surgical approach to disease was not in line with the patient’s own stated goals for care after a balanced risk/benefit discussion.

Finally, the number of patients that undergo DP-CAR is a heavily selected subset of patients that present with concern for celiac axis involvement. The denominator (all patients presenting with localized disease and potential involvement of the CA to our multi-disciplinary clinic) is challenging to capture in our surgical database and due in part to our routine in preoperative planning and logistics. Many of these DP-CAR patients are taken to the operating room with a plan to either complete standard DP or DP-CAR as guided by intraoperative assessment of the tumor in relation to the celiac and its branches. In addition, many patients who are captured in our database as ‘straightforward’ DP were originally taken to the OR with a plan for DP-CAR but with DP completed due to intraoperative findings. In this group we do not have preoperative plans (i.e. a plan for DP-CAR) detailed in our database and, as such, we do not know the total number of patients who were prepped for potential DP versus DP-CAR.

Despite these limitations, using the largest single center Western cohort, this is the first series to examine the surgery specific complication rates in DP-CAR and to examine the patterns of recurrence, both of which appear to be different than patients undergoing distal pancreatectomy without celiac axis resection. Future prospective study with capture of patients who do not undergo surgery looking at disease specific outcomes and patterns of recurrence will be required in the context of continued aggressive induction chemotherapy.

## Conclusion:

Aggressive multi-agent induction chemotherapy and radiation therapy have resulted in the recognition of a patient cohort with locally confined advanced pancreatic cancer amenable to margin negative resection with DP-CAR. These patients were historically considered to have unresectable disease and dismal overall survival. This surgical approach appears to have an acceptable postoperative complication profile, with unique procedure specific complications (gastric ischemia and chyle leak), as well as a unique long-term pattern of recurrence with lower than expected liver only disease. Close monitoring of the safety and oncologic benefit in this subgroup of patients with locally advanced pancreatic cancer is required as aggressive surgical therapy is more frequently employed in the service of these patients.

## Figures and Tables

**Table 1 –:** Demographics:

Demographics	
Age, years, median (IQR)	62.7 (57.0–68.0)
Gender, % male (n)	46.3% (25)
BMI, kg/m^2^, median (IQR)	25.6 (22.2–27.2)
Race % (n)	
Caucasian	85.2% (46)
African American	10.9% (5)
Other	4.4% (2)
ASA Class, % (n)	
I	0.0% (0)
II	20.4% (11)
III	72.2% (39)
IV	1.9% (1)
Co-Morbidities, % (n)	
Hypertension	48.2% (26)
Diabetes Mellitus	16.7% (9)
Coronary Artery Disease	7.4% (4)
Chronic Kidney Disease	1.9% (1)
Smoking History, % (n)	
Current Smoking	11.1% (6)
Past Smoking	11.1% (6)
Current Moderate Alcohol Use, % (n)	18.5% (10)
Preoperative Characteristics	
Presenting Symptoms, % (n)	
Weigh Loss	42.6% (23)
Abdominal Pain	77.8% (42)
Neoadjuvant Chemotherapy, % (n)	98.2% (51)
FOLFIRINOX	70.4% (38)
Gemcitabine/Abraxane	20.4% (11)
Other	7.4% (4)
Duration of Neoadjuvant Therapy, months, median (IQR)	4.0 (4.0–6.0)
Neoadjuvant Radiotherapy, % (n)	94.4% (51)
SBRT	74.1% (40)
Standard RT	20.3% (11)
Laboratory Values, median (IQR)	
CA 19–9 (U/ml)	68.9 (34.1–359.9)
Bilirubin (mg/dL)	0.4 (0.3–0.6)
Creatinine (mg/dL)	0.8 (0.7–0.9)
Albumin (g/dL)	4.2 (3.9–4.5)

**Table 2 –:** Operative Characteristics:

Postoperative Characteristics	
Length of Stay, days, median (IQR)	8.0 (6.0–9.0)
Operative Time, min, median (IQR)	371.0 (281.0–432.0)
EBL, mL, median (IQR)	750 (400–1000)
Transfused Postoperatively, % (n)	29.6% (16)
Arterial Reconstruction, % (n)	16.7% (9)
Celiac/Aorta to CHA (n)	6
Iliac to CHA (n)	1
Renal to CHA (n)	1
Iliac to right and left HA (n)	1
Multi-visceral Resection, % (n)^1^	29.6% (16)
Partial Gastrectomy (n)	7
Total Gastrectomy (n)	3
Adrenalectomy (Partial or Total) (n)	7
Nephrectomy (n)	1
Liver Wedge Resection (n)	1
Postoperative Day 1 Labs	
ALT (U/L)	96.0 (42.0–239.0)
AST (U/L)	104.0 (55.0–175.0)
Pathologic Characteristics	
Node Positive, % (n)	35.2% (19)
Number of Nodes, median (IQR)	20.5 (16.0–29.0)
Positive Lymph Node Ratio, median (IQR)	0.07 (0.05–0.1)
Margin Positive (R1)	13.0% (7)
Tumor size, cm, median (IQR)	3.0 (2.2–4.0)
Lymphovascular Invasion, % (n)	24.1% (13)
Perineural Invasion, % (n)	57.4% (31)
Tumor Grade, % (n)	
Well Differentiated	1.9% (1)
Moderately Differentiated	57.4% (31)
Poorly Differentiated	24.1 (13)
Significant Treatment Effect	11.1% (6)
Treatment Response Reported, % (n)	83.3% (45)
Grade 1 (near complete response)	31.1% (14)
Grade 2 (partial response)	51.1% (23)
Grade 3 (poor or no response)	17.8% (8)

1. Numbers sum to more than 11 because several patients had multiple organ resections, CHA = common hepatic artery

**Table 3 –:** Postoperative Complications:

Postoperative Complications, % (n)	
Overall Complication Rate	42.6% (23)
Clavien-Dindo 3a-4b	18.5% (10)
Clinically Relevant Postoperative Pancreatic Fistula	9.3% (5)
Delayed Gastric Emptying (Grade B/C)	7.4% (4)
Post-pancreatectomy Hemorrhage	1.9% (1)
Chyle Leak	16.7% (9)
Gastric Perforation or Ischemia	3.7% (2)
Reoperation	1.9% (1)
30-day readmission	27.8% (15)
90-day readmission	35.2% (19)
30-day mortality	0.0% (0)
90-day mortality	1.9% (1)

**Table 4 –:** Recurrences:

First Site of Recurrence, % (n)	
No Recurrence	25.9% (14)
Local Only	35.1% (19)
Lung Only	13.0% (7)
Peritoneal	11.1% (6)
Local and Distant	7.4% (4)
Liver Only	7.4% (4)

## Synopsis:

Aggressive surgical therapy for locally advanced pancreas cancer with distal pancreatectomy and celiac axis resection is associated with unique procedure specific complications and patterns of recurrence. With careful patient selection, it appears safe with favorable oncologic outcomes.
